# 2-Methyl-2,4-pentanediol (MPD) boosts as detergent-substitute the performance of ß-barrel hybrid catalyst for phenylacetylene polymerization

**DOI:** 10.3762/bjoc.13.148

**Published:** 2017-07-31

**Authors:** Julia Kinzel, Daniel F Sauer, Marco Bocola, Marcus Arlt, Tayebeh Mirzaei Garakani, Andreas Thiel, Klaus Beckerle, Tino Polen, Jun Okuda, Ulrich Schwaneberg

**Affiliations:** 1 Institute of Biotechnology, RWTH Aachen University, Worringer Weg 3, 52074 Aachen, Germany; 2 Institut für Anorganische Chemie, RWTH Aachen University, Landoltweg 1, 52074 Aachen, Germany; 3 DWI - Leibniz Institute for Interactive Materials e.V., Forckenbeckstr. 50, 52056, Aachen, Germany; 4 Institute of Bio- und Geosciences, IBG-1: Biotechnology, Forschungszentrum Jülich GmbH, 52425 Jülich, Germany

**Keywords:** amphiphilic molecule 2-methyl-2,4-pentanediol, hybrid catalyst, phenylacetylene polymerization, refolding agents, transmembrane protein FhuA

## Abstract

Covering hydrophobic regions with stabilization agents to solubilize purified transmembrane proteins is crucial for their application in aqueous media. The small molecule 2-methyl-2,4-pentanediol (MPD) was used to stabilize the transmembrane protein *Ferric hydroxamate uptake protein component A* (FhuA) utilized as host for the construction of a rhodium-based biohybrid catalyst. Unlike commonly used detergents such as sodium dodecyl sulfate or polyethylene polyethyleneglycol, MPD does not form micelles in solution. Molecular dynamics simulations revealed the effect and position of stabilizing MPD molecules. The advantage of the amphiphilic MPD over micelle-forming detergents is demonstrated in the polymerization of phenylacetylene, showing a ten-fold increase in yield and increased molecular weights.

## Introduction

The combination of a transition metal catalyst and a protein by either dative, supramolecular or covalent means leads to so-called artificial metalloenzymes or biohybrid catalysts [1–2]. Using a non-natural catalyst, the scope of natural enzymes can be expanded or the activity improved. Recent examples are the construction of metatheases [3–4], asymmetric transfer hydrogenases [5–6], Diels-Alderases [7–10], an enzyme for carbon–silicon bond formation [11], a phenylacetylene polymerase [12–13] and others [14–17].

A challenge to overcome are unintended substrate–protein interactions, e.g., repulsion of polar substrates with polar amino acid residues [18]. Furthermore, nonpolar substrates are poorly soluble in water and often build a second phase or require a cosolvent. For proteins, these conditions are challenging. The interaction of solvents with the protein can destroy the three dimensional structure and cause protein precipitation [19–21]. To avoid precipitation when using nonpolar substrates, the protein concentration usually is decreased leading to a loss in activity. As an example, the polymerization of phenylacetylene was achieved in water by using the robust β-barrel protein nitrobindin. The selectivity in the polymerization of phenylacetylene was influenced with the protein as second ligand sphere [12–13]. The catalyst achieved a *cis*/*trans* ratio of 91:9 in the organic solvent tetrahydrofuran (THF) or being bound on a protein surface without a defined protein environment [12]. By mutations within the cavity of the protein, the ratio was almost inverted to *cis*/*trans* 18:82 [13]. Nevertheless, the productivity remained low due to the decreased protein concentration.

A strategy to increase the stability of proteins is the use of whole-cell catalysts. Cells usually show increased stability towards cosolvents, pH and elevated temperatures [22–23]. A recent example in the field of artificial metalloenzymes was shown by Ward and co-workers, who used an artificial metathease in an in vivo approach. These first attempts are promising to generate artificial whole-cell catalysts. Nevertheless, the productivity with a turnover number of 6 (with respect to the metal content) is yet low [4].

Here, we present a new strategy based on the robust β-barrel protein *Ferric hydroxamate uptake protein component A* (FhuA, *T*_m_ 60–65 °C, refolding after heating up to 85 °C, THF up to 40 vol % tolerated) [19,24–27]. FhuA is one of the largest known outer membrane proteins consisting of 22 antiparallel β-sheets, which are connected through long extracellular loops and short periplasmic turns. After removal of the barrel-plugging “cork” domain (Δ1-160), the formed pore (2.5–3.0 nm) is sufficiently large to harbor sterically demanding catalysts and substrates [28–29]. As a transmembrane protein, FhuA needs stabilization of its hydrophobic transmembrane region in an aqueous environment, which is naturally covered by phospholipids in the outer membrane of *Escherichia coli* (*E. coli*) [30]. For extraction of membrane proteins, commonly micelle-forming detergents such as sodium dodecyl sulfate (SDS), polyethylene–polyethyleneglycol (PE–PEG), sugar glycosides or polyoxyethylenes are applied [24–25,28,31–32]. SDS is an efficient detergent for membrane protein solubilization, but is leading to protein unfolding as a drawback. Disadvantageous of detergents is the tremendous reduction of selectivity due to denaturing the protein or the reduction of productivity by detergent micelles since hydrophobic compounds are most likely located inside the hydrophobic micelle core. Recently, the small amphiphilic alcohol 2-methyl-2,4-pentanediol (MPD) was shown to successfully stabilize membrane proteins and enable characterization of protein modifications [33–34]. Polymerization of phenylacetylene in the presence of MPD molecules as refolding agent was carried out, reaching higher molecular weights and yields compared to catalysis with the micelle-forming refolding reagent PE–PEG. Minimum of MPD molecules was analyzed by molecular dynamics studies to enable refolding of SDS-denatured transmembrane protein FhuA ΔCVF^tev^ [29].

This report aims to demonstrate the importance of the right choice of the membrane protein stabilizer for biohybrid catalysis.

## Results and Discussion

**Figure 1:**
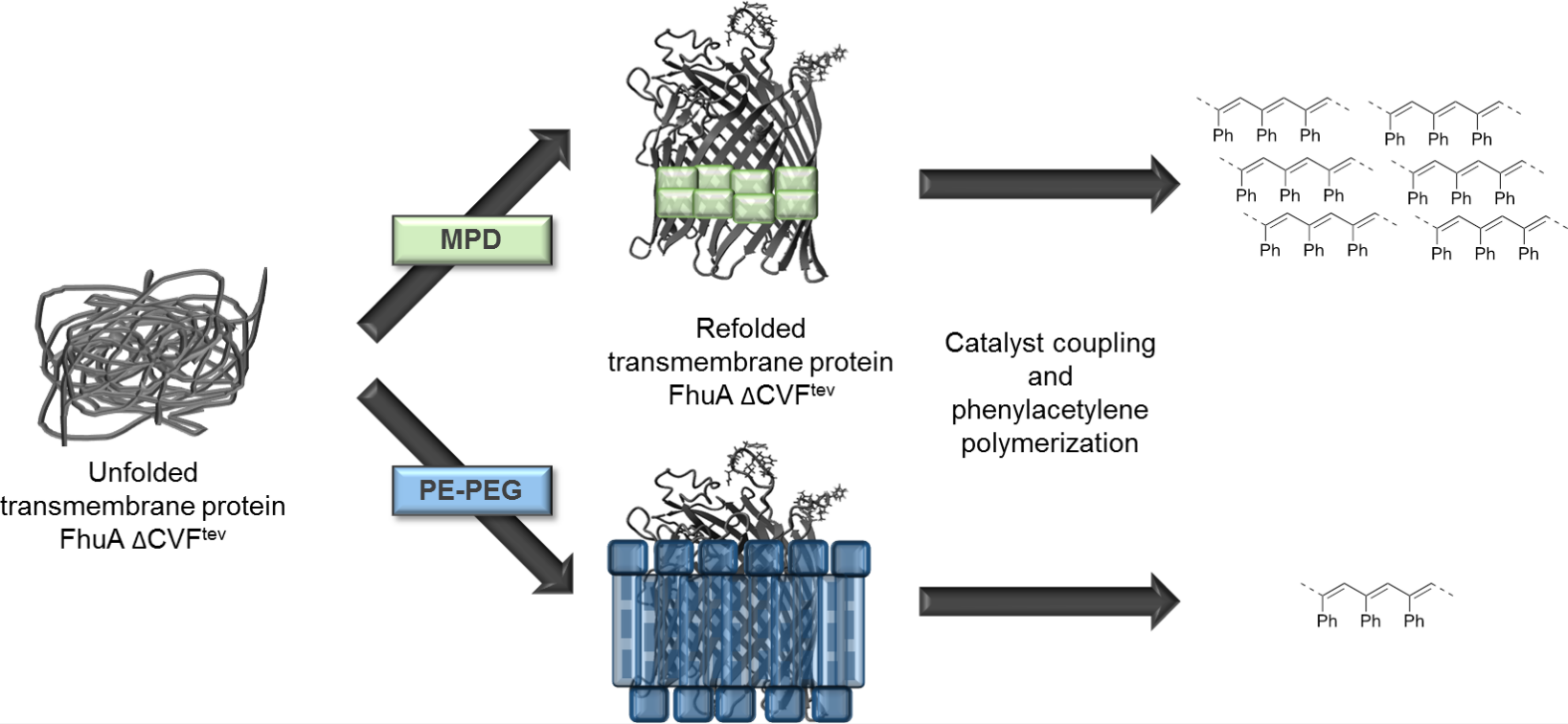
Phenylacetylene polymerization of FhuA ΔCVF^tev^ [29] refolded in a polymer or small amphiphilic molecule. Refolding agents are essential to solubilize transmembrane proteins and keep membrane proteins refolded by shielding hydrophobic residues in aqueous environments. Refolding of the open channel protein FhuA ΔCVF^tev^ was on one hand achieved with polyethylene–polyethyleneglycol (PE–PEG), which is a micelle-forming detergent. In contrast, refolding of FhuA ΔCVF^tev^ with 2-methyl-2,4-pentanediol (MPD) prevents micelle formation and leads to increased yield and molecular weight of the corresponding polymer.

For solubilizing the transmembrane protein FhuA ΔCVF^tev^ PE–PEG and MPD were applied as stabilizing agent and phenylacetylene polymerization was performed as model reaction (Figure 1).

### Molecular dynamics (MD) simulations reveal an optimal minimum number of ≈200 MPD molecules for shielding the hydrophobic transmembrane region of FhuA ΔCVF^tev^

**Figure 2:**
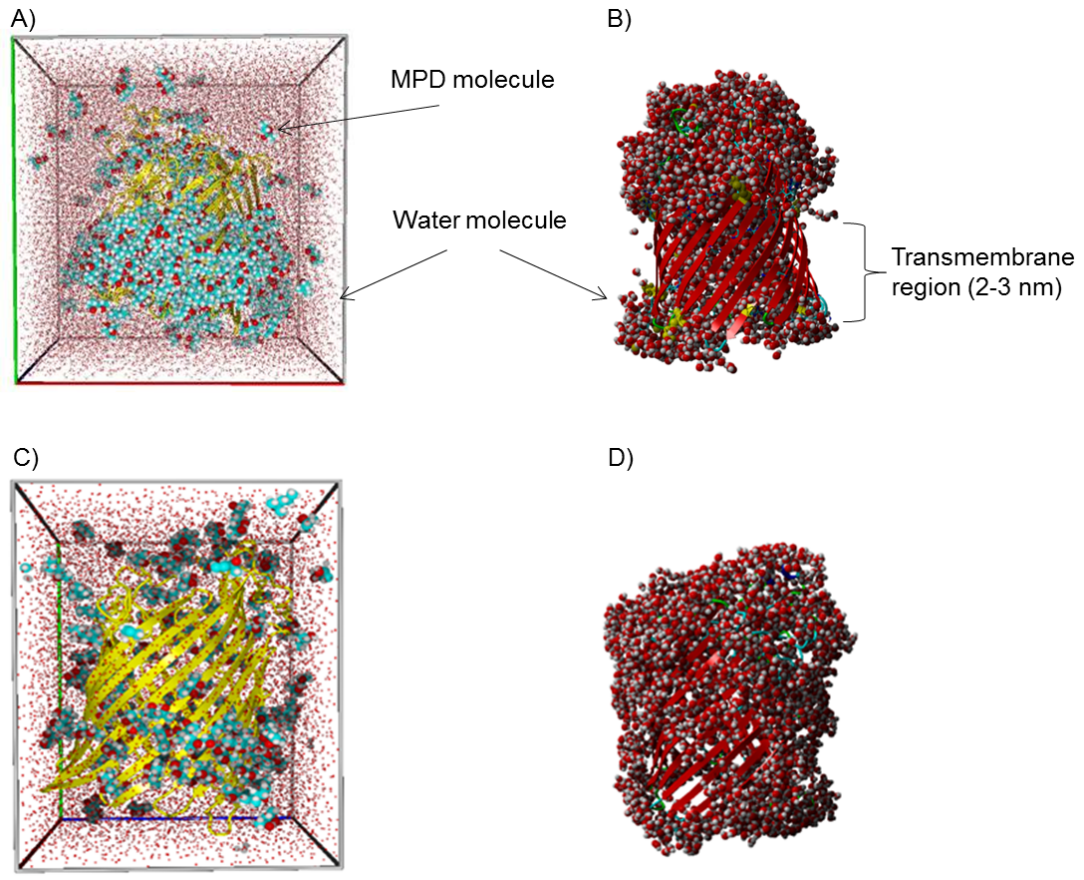
Hydrophobic transmembrane region of FhuA ΔCVF^tev^ [29] stabilized by ≈200 MPD molecules. MPD is illustrated as mainly cyan molecules, water molecules are mainly red. A) A belt of 209 MPD molecules is located close to the transmembrane area. FhuA ΔCVF^tev^ with 22,374 water and 378 MPD molecules was used as starting condition, in which most MPD molecules diffused away. B) Water molecules in the first solvation sphere (<5 Å) of FhuA ΔCVF^tev^ are shown to visualize that the transmembrane area of FhuA ΔCVF^tev^ is completely water free in MD simulations using 378 MPD molecules. C) MD simulations of FhuA ΔCVF^tev^ with 12,208 water and 126 MPD molecules show that a saturation of the transmembrane region could not be achieved, leading to an incomplete coverage of the hydrophobic belt. D) Water molecules in the first solvation sphere (<5 Å) are partly covering the hydrophobic belt of FhuA ΔCVF^tev^ using 126 MPD molecules. MPD, 2-methyl-2,4-pentanediol.

MD simulations of FhuA ΔCVF^tev^ were performed in a box with varying numbers of MPD molecules from 126 MPD, 189 MPD, 252 MPD to 378 MPD molecules as stabilizing cosolvent to investigate the molecular dynamics of protein structure stabilization, how a small amphiphilic molecule could stabilize a transmembrane protein such as FhuA ΔCVF^tev^. All simulations started with a random distribution of MPD, but after a few nanoseconds, the MPD molecules start to cluster around the hydrophobic transmembrane region. Membrane proteins are normally stabilized by incorporation in a protecting membrane layer formed by ionic detergent molecules such as lipids, SDS or nonionic glycolipids. In contrast, in MD simulations with the two highest concentrations MPD forms a small layer of around 200 MPD molecules. The layer is completely covering the transmembrane region and forms a soluble complex, as can be seen in Figure 2A and B). Using less MPD molecules leads to an insufficient coverage (Figure 2C and D) and thus less stabilization of the membrane protein FhuA ΔCVF^tev^. The theoretical calculations are in line with the experimental findings, that FhuA ΔCVF^tev^ is properly folded using refolding buffer with 50 mM MPD, which was confirmed by CD spectroscopy (Figure S1, Supporting Information File 1).

### 2-Methyl-2,4-pentanediol stabilizes FhuA ΔCVF^tev^ up to eight weeks

Keeping membrane proteins properly folded outside of a biological membrane is a challenging task. Detergents are needed to refold the applied membrane proteins after their extraction from the natural bilayer environment [35–40]. In case of FhuA, so far, refolding has been reported by protecting its hydrophobic transmembrane region in the presence of a detergent such as octylpolyoxyethylene (oPOE) or block copolymer such as PE–PEG [19,28,31,41–44]. Although PE–PEG improves protein solubility, polymerization reactions utilizing FhuA ΔCVF^tev^ as protein host in the presence of this copolymer go along with losses in yield due to its micelle-forming property, leading to the need for other types of detergents. Therefore, using a small amphiphilic molecule as an alternative to polymeric detergents is desirable in order to overcome this limitation (Table 1).

**Table 1:** Comparison of common solubilizing agents for membrane proteins.^a^

Refolding agent	Activity	Selectivity	Comment

SDS [33]	++	−	unfolding property
oPOE [19,29]	+	+	costly, micelle formation

PE–PEG [29]	+	++	bulky, micelle formation
MPD [33–34]	++	++	small, amphipathic alcohol, water-miscible

^a^SDS, sodium dodecyl sulfate; oPOE, octylpolyoxyethylene; PE–PEG, polyethylene–polyethyleneglycol; MPD, 2-methyl-2,4-pentanediol. ++, very good; +, beneficial; −, non-beneficial.

In this study, we used the water-miscible amphipathic alcohol MPD (118.18 g/mol) as stabilizing agent in addition to the commonly used PE–PEG [39,45]. The method, originally developed by Michaux and colleagues, consists of using amphipathic cosolvents to refold SDS-denatured proteins and enable them to regain their 3D structure [33,46]. Using MPD is not only beneficial for the polymerization process, but also enables the use of characterization techniques such as transmission electron microscopy and atomic force microscopy. Polymeric detergents are effective protein-stabilizing agents mainly at high concentrations. In contrast, the polymerization using FhuA ΔCVF^tev^ could be achieved at lower millimolar concentrations of MPD, which binds tightly to the channel protein. A buffer containing 50 mM MPD was used in the experiments, which contains more than 3 times of the minimum required value for FhuA ΔCVF^tev^ (see MD simulation results, Figure 2C and D), ensuring the long-term stability of the protein (Figure S1, Supporting Information File 1). The aforementioned features are consistent with results from circular dichroism (CD) spectroscopy (Figure S1, Supporting Information File 1), showing that FhuA ΔCVF^tev^ is correctly folded even up to eight weeks.

### Coupling efficiency of the rhodium catalyst to FhuA ΔCVF^tev^ is more than 90%

**Scheme 1:**
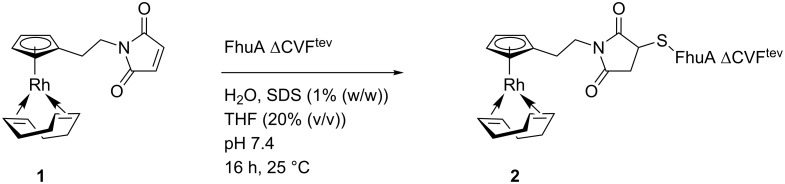
Coupling of [Rh]-**1** to the open channel protein FhuA ΔCVF^tev^. SDS, sodium dodecyl sulfate; THF, tetrahydrofuran.

The rhodium catalyst **1** bearing a maleimide group was attached to FhuA ΔCVF^tev^ for the generation of the biohybrid catalyst [Rh]-FhuA ΔCVF^tev^ **2** as previously reported for the Grubbs–Hoveyda type [29,47] or copper complexes [10] (Scheme 1).

**Figure 3:**
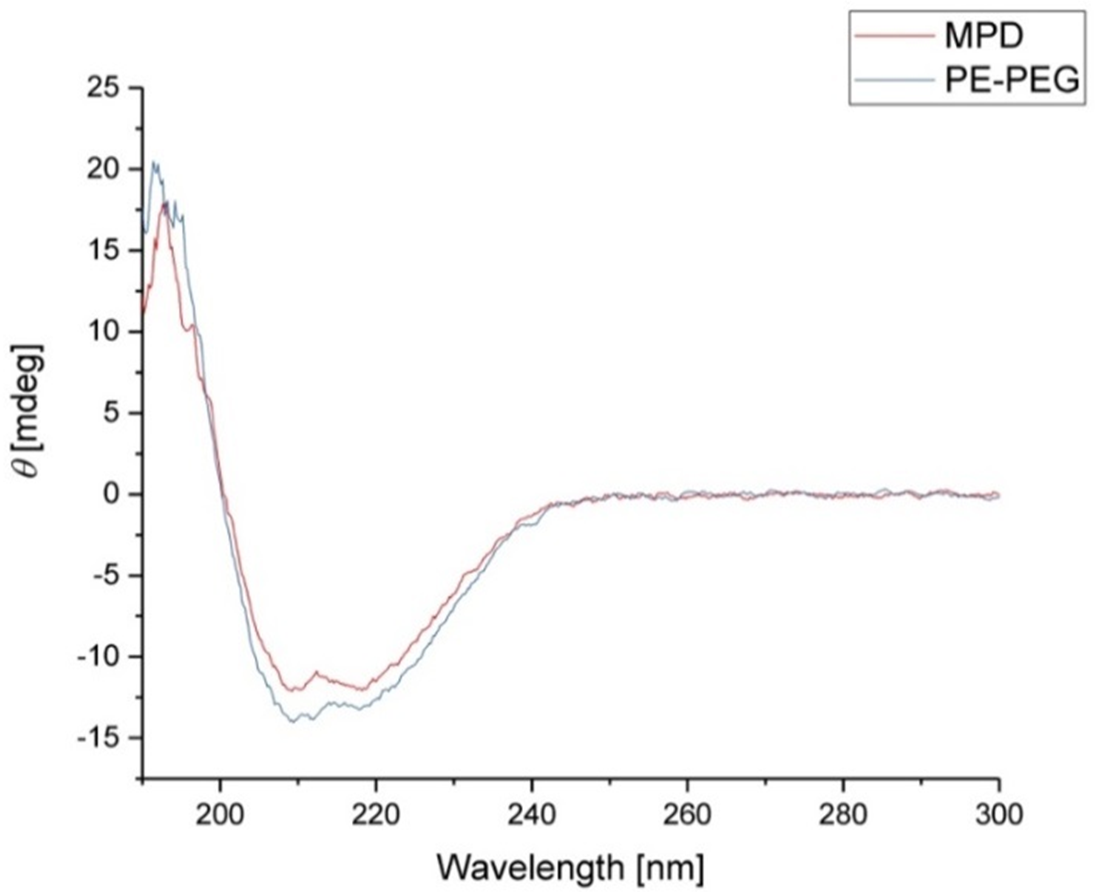
Circular dichroism spectra of **2** refolded in 2-methyl-2,4-pentanediol (MPD, red) and polyethylene–polyethyleneglycol (PE–PEG, blue).

FhuA ΔCVF^tev^ was dissolved in a solution containing 1.25% SDS. The state of FhuA ΔCVF^tev^ is partially unfolded. The catalyst **1** easily accesses the thiol group (Cys545, numbering based on FhuA WT with PDB ID 1BY3 [24]) introduced for maleimide thiol coupling and a high coupling efficiency is achieved (Figure S2, Supporting Information File 1). After coupling, the excess catalyst is removed by washing the protein residue with THF. The dried biohybrid conjugate **2** is dissolved in water and refolded. As refolding reagents, the block copolymer PE–PEG and amphiphilic MPD are used, respectively. Refolding is achieved by dialysis of the protein in a solution containing the particular refolding agents. The structural integrity of FhuA ΔCVF^tev^ was confirmed with CD spectroscopy (Figure 3).

When either PE–PEG or MPD is applied, the CD spectra for the biohybrid conjugate **2** show typical features of a β-barrel structure (maximum around 195 nm, minimum around 215 nm) [48], indicating a successful refolding of the transmembrane protein FhuA ΔCVF^tev^ with both reagents.

**Figure 4:**
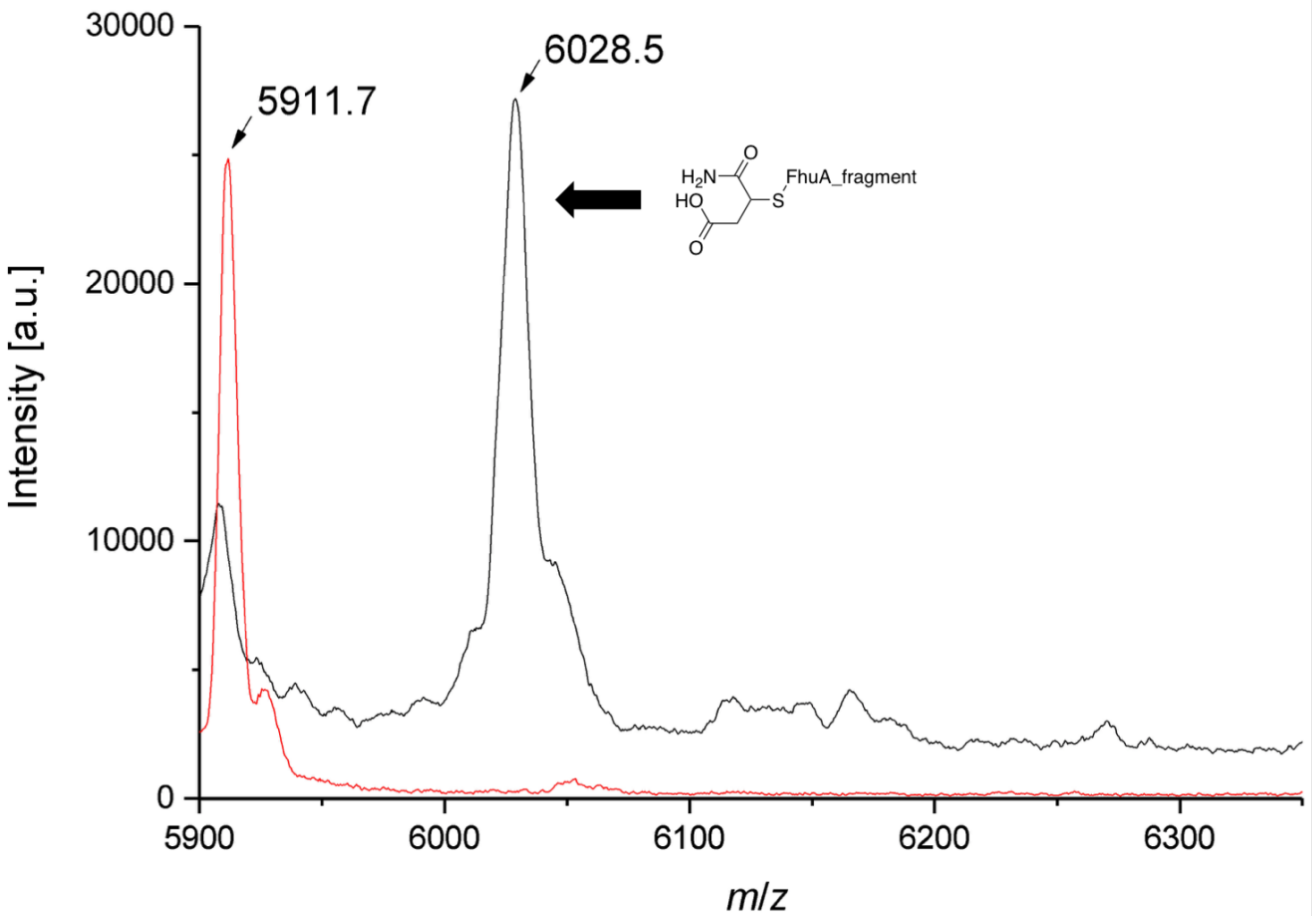
MALDI–TOF mass spectra of apo FhuA ΔCVF^tev^ (red; calculated *m*/*z* = 5902.6; found: *m*/*z* = 5911.7) and **2** (black; *m*/*z* = 6028.5 is assigned to the FhuA fragment containing the maleimide function after water addition). Possible fragmentation of the [Rh] catalyst is indicated. FhuA ΔCVF^tev^ was analyzed after digestion by protease from Tobacco Etch Virus (TEV).

The coupling efficiency was determined by fluorescence titration of the cysteine function of **2** (Cys545) using the fluorescence dye ThioGlo^®^ 1 (fluorescent thiol reagent, Figure S2, Supporting Information File 1). More than 90% of the cysteines are occupied, showing a very high coupling efficiency of the rhodium catalyst. Further, the biohybrid conjugate was analyzed by MALDI–TOF mass spectrometry prior to digestion of **2** with the protease of the Tobacco Etch Virus (TEV) [29,47,49]. Even though the calculated mass of 6,301 Da for the FhuA ΔCVF^tev^ fragment containing Cys545 and the metal catalyst (≈6 kDa) could not be observed, the MALDI–TOF mass spectra indicate the successful conjugation of the catalyst by an increase of the molecular weight of 116 Da corresponding to the maleimide group (Figure 4). In studies with other catalysts attached to FhuA ΔCVF^tev^, the addition of water to the maleimide ring was observed [10,47]. During digestion or ionization, also cleavage of the amide bond occurs and therefore the metal cannot be observed.

### Polymerization of phenylacetylene

The synthesized and characterized biohybrid conjugate based on FhuA ΔCVF^tev^ was used to polymerize phenylacetylene (**3,** Table 2).

**Table 2:** Results of phenylacetylene (**3**) polymerization catalyzed by biohybrid conjugate **2**.^a^

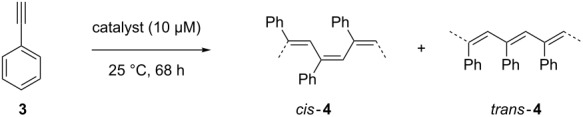						

Entry^b^	Catalyst	Stabilization agent^c^	Isolated yield (%)	*M*_n_^d^ (g/mol)	PDI^d^	*trans*/*cis*^e^

1^f^	**1**		13 mg (65)	5,300	4.6	10:90

2^g^	FhuA ΔCVF^tev^	PE–PEG or MPD	–	–	–	–
3^g^	**2**	PE–PEG	<1 mg (5)	800	6.0	70:30

4^g^	**2**	MPD	10 mg (52)	5,500	2.9	75:25

^a^THF, tetrahydofuran, PE–PEG, polyethylene–polyethylene glycol; MPD, 2-methyl-2,4-pentanediol. ^b^Buffer: Water containing NaP_i_ (pH 8, 10 mM) and EDTA (1 mM). *c*(**3**) = 0.1 M; *V*_total_ = 2 mL. ^c^*c*(PE–PEG) = 0.125 mM; *c*(MPD) = 50 mM. ^d^Determined by GPC. ^e^Determined with ^1^H NMR spectroscopy. ^f^Reaction in THF. ^g^Reaction in buffer, containing 10% (v/v) THF.

Polymerization of phenylacetylene in THF at 25 °C yields in 65% polymer with *M*_n_ = 5,300 and a high *cis*-content of 90% (Table 2, entry 1). If the rhodium catalyst is not present, FhuA ΔCVF^tev^ itself is not able to convert the substrate, as expected (Table 2, entry 2). The polymerization reaction of **3** with the biohybrid catalyst **2** is strongly dependent on the choice of the stabilization reagent. In case of PE–PEG, FhuA ΔCVF^tev^ precipitation is observed. Filtering of the solution shows similar results as the reaction with the precipitate present, indicating a deactivation of the catalyst or restricted access of the substrate to the active site. The isolated polymer yield is approximately 5% (Table 2, entry 3). Polymer analysis with gel permeation chromatography (GPC) shows only an oligomeric fraction (*M*_n_ up to 800 g/mol). Applying the refolding reagent MPD, the solution stays clear and turns turbid over time. The yellow to orange color indicates successful polymer formation. The isolated polymer was analyzed by GPC, showing a nearly seven-fold increased molecular weight (*M*_n_ = 5,500) compared to the polymerization reaction with PE–PEG (Table 2, entry 4). Further, the isolation is easier due to facilitated removal of the MPD compared to the polymeric refolding reagent PE–PEG. The isolated yield increased from 5% to 52%. This is related to the increased FhuA ΔCVF^tev^ stability in the presence of hydrophobic substrates. The hydrophobic phenylacetylene interacts with the micelles formed by the PE–PEG refolding reagent, causing the protein precipitation. Experiments utilizing dynamic light scattering (DLS) revealed an interaction of the PE–PEG micelles with the phenylacetylene, showing a decrease in the size distribution of micelles (Figure S3, Supporting Information File 1). Increasing the MPD concentration (up to *c*(MPD) = 200 mM) did not lead to a significant increased polymer yield.

The selectivity of the polymerization was affected by the FhuA scaffold. Due to the fact that a catalyst is covalently attached inside of a protein scaffold and surrounded by amino acid residues. The protein free catalyst **1** shows a high *cis*-selectivity (90%). The biohybrid conjugate almost inverts the selectivity, showing 70% *trans*-selectivity independent from the choice of detergent (Table 2, entry 3 and entry 4). Based on the results of *cis/trans* ratios not detergents, but the FhuA scaffold leads to changes in selectivity and emphasizes the position of the catalyst inside the barrel. Similar findings were made by Hayashi and co-workers, utilizing the soluble protein nitrobindin as protein scaffold. Upon anchoring of the catalyst to the nitrobindin mutant, the selectivity drastically changed. Further, the group gradually influenced the selectivity by changing the direct environment of the catalyst by introducing sterically demanding amino acids in the protein cavity [13].

Additionally, FhuA ΔCVF^tev^ is stable over the time. As reported by Hayashi and co-workers, the polymerase based on nitrobindin loses structural integrity after 12 hours, resulting in a loss of *cis*/*trans* selectivity [13]. The membrane protein FhuA ΔCVF^tev^ in MPD shows stability for more than three days under the reaction conditions and therefore is leading to significantly increased yields.

## Conclusion

In conclusion, we successfully demonstrated the use of MPD as small-molecule stabilizer for utilization of the biohybrid catalyst **2** in phenylacetylene polymerization. The small detergent MPD stabilizes the transmembrane protein FhuA ΔCVF^tev^ in aqueous solution without forming micelles. The structural integrity was proven by CD spectroscopy. Applying MPD as stabilizing agent, an approximately ten-fold increase in yield of poly(phenylacetylene) was obtained compared to reactions in PE–PEG containing solutions. MD simulations revealed the refolding-supporting behavior of the MPD molecules shielding the hydrophobic transmembrane regions of FhuA ΔCVF^tev^.

This finding makes the use of membrane proteins more attractive. When using other stabilizing agents, micelle formation decreases the activity by building up an additional diffusion barrier. Furthermore, the formed micelles are influenced by the substrate leading to protein precipitation. The usage of the amphiphilic stabilizer MPD avoided protein precipitation leading to increased yields.

The membrane protein FhuA is robust towards external influences such as increased temperatures and pH values. The catalyst and substrate scope in biohybrid catalysis can be fine-tuned choosing a suitable stabilizing agent as shown in this report. These results may inspire the tailoring of membrane proteins as catalysts in the field of biohybrid catalysis.

## Experimental

### General comments

All used chemicals used were of analytical grade or higher quality, purchased from Applichem (Darmstadt, Germany) or Sigma-Aldrich Chemie (Taufkirchen, Germany).

All operations were performed under an inert atmosphere of argon or nitrogen using standard Schlenk or glove box techniques if not mentioned otherwise. Water and other solvents were degassed by using the “freeze-pump-thaw” technique. THF was obtained dry and degassed from a SPS 800 from MBraun (Garching, Germany). Chloroform-*d*_1_ was dried over calcium hydride, distilled, degassed and stored in a glove box. NMR spectra were recorded on a Bruker DRX 400 spectrometer (^1^H, 400.1 MHz). Chemical shifts were referenced internally by using the residual solvent resonances [50]. MALDI–TOF MS spectra were recorded on an Ultraflex III TOF/TOF mass spectrometer (Bruker Daltonics, Billerica, MA, US). GPC was measured on an Agilent Series 1100 (Midland, ON, Canada), equipped with two SDV linear N columns of 8 × 300 mm and 8 × 600 mm measures and 5 µm pore size, in THF at 30 °C against a poly(styrene) standard. Dynamic light scattering was performed with a Zetasizer Nano Line (Malvern Instruments, Worcestershire, UK). The rhodium catalyst **2** was synthesized according to literature procedures [12]. Phenylacetylene (**3**) is commercially available and was used as received. All other chemicals were used as received if not mentioned otherwise.

### Expression and extraction of FhuA ΔCVF^tev^

Expression of FhuA ΔCVF^tev^ from T7 expression vector pPR-IBA1 was performed using the *E. coli* B^E^ BL21 (DE3) omp8 strain as expression host according to previous descriptions [29,34,51]. FhuA ΔCVF^tev^ was extracted from *E. coli* with SDS as solubilizing agent as described previously [19,29,34]. Refolding of FhuA ΔCVF^tev^ in 1.25% SDS was performed by dialysis against 0.125 mM PE–PEG or 50 mM MPD, respectively [29,34]. Protein concentration was determined by bicinchoninic acid reaction (PierceTM BCA Protein Assay Kit, Thermo Fisher Scientific, Darmstadt, Germany). Refolding buffers are defined as 10.0 mM sodium phosphate buffer pH 8.0 and 1.0 mM EDTA with the addition of 0.125 mM PE–PEG (PE–PEG buffer) or 50.0 mM MPD (MPD buffer) for the purpose of this article.

### Cleavage of FhuA ΔCVF^tev^ with TEV protease

For analysis of the modification of Cys545 of FhuA ΔCVF^tev^ with MALDI–TOF mass spectrometry, two cleavage sites of the TEV protease (ENLYFQ|G) were introduced in the extracellular loop regions 7 and 8 [29]. Protease cleavage was performed as described previously [12,29].

### MD simulations

Simulations were based on the X-ray crystal structure of the β-barrel membrane channel protein FhuA WT co-crystallized with the detergent *n*-octyl-2-hydroxyethyl sulfoxide [24]. The N-terminal cork domain (residue 1-160) blocking the channel was removed. The amino acid exchanges of the hybrid catalyst model FhuA ΔCVF^tev^, namely cysteine at position 545, valine at position 548, phenylalanine at position 501 and two flanking TEV-protease recognition sequences in loop 7 and loop 8 were introduced using YASARA Structure 13.6.13 as described previously [29] in a detergent-membrane model stabilized by octylpolyoxyethylene (*n* = 5). To study the interactions of the membrane protein variant FhuA ΔCVF^tev^ with the amphiphilic stabilizing agent MPD, FhuA ΔCVF^tev^ was solvated in a periodic box (size 79.57 × 89.35 × 64.87 or 95.49 × 82.38 × 105.82 with α, β and γ = 90.00°) filled with 12,208 or 22,374 TIP3P water molecules and 126, 189, 252 or 378 randomly distributed MPD molecules as cosolvent [52–56]. The MD calculations (75 ns each) were performed using the AMBER99 force field for the protein and GAFF for MPD cosolvent. The electrostatic interactions were calculated using a 8 Å cut-off and Particle Mesh Ewald [57] for long range electrostatics at pH 7.4 and a density of 0.997 g/mL. The hydrophobic membrane area was covered by an average of 200 MPD molecules in the last 10 ns of the MD simulations, avoiding direct water contact.

### Coupling and purification

To a degassed solution of FhuA ΔCVF^tev^ (5–6 mg/mL) in aqueous SDS solution (1% (w/w) SDS, pH 8 adjusted with NaHCO_3_), rhodium catalyst **2** (10 equiv) in THF (10% (v/v)) was added dropwise. The mixture was stirred for 16 h at room temperature. The solvent was removed in vacuo and the residue washed with THF (3 × 15 mL). The residue was dried in vacuo and dissolved in water. Refolding of the biohybrid conjugate was achieved as described above for the apo protein.

### Polymerization of phenylacetylene

To an aqueous solution of refolded **2** (2 mL, 10 µM, refolded with either PE–PEG or MPD) in air atmosphere, phenylacetylene (**3**) in THF (10% (v/v) THF, final concentration of **3** = 0.1 M) was added. The mixtures were stirred at room temperature. After the appropriate reaction time, the polymer was extracted with chloroform, dried in vacuo, washed with water and analyzed by ^1^H NMR and GPC as reported previously [12–13].

## Supporting Information

File 1 CD spectra of unmodified FhuA ΔCVF^tev^ directly after refolding and after eight weeks, Thioglo^®^ 1 titration and DLS results.
