# Supplementary material for: 2-Methyl-2,4-pentanediol (MPD) boosts as detergent-substitute the performance of ß-barrel hybrid catalyst for phenylacetylene polymerization
Source: Beilstein J Org Chem. 2017 Jul 31;13:1498–506. doi: 10.3762/bjoc.13.148 (PMC5550818; doi:10.3762/bjoc.13.148)
Supplement: File 1 — CD spectra of unmodified FhuA ΔCVFtev directly after refolding and after eight weeks, Thioglo® 1 titration and DLS results. [file Beilstein_J_Org_Chem-13-1498-s001.pdf]

Supporting Information

for

2-Methyl-2,4-pentanediol (MPD) boosts as detergent-substitute the performance of ß-barrel hybrid catalyst for phenylacetylene polymerization

Julia Kinzel[1#], Daniel F. Sauer[2#], Marco Bocola[1], Marcus Arlt[1], Tayebeh Mirzaei Garakani[1,3], Andreas Thiel[2], Klaus Beckerle[2], Tino Polen[4], Jun Okuda[2] and Ulrich Schwaneberg[1,3*]

Address: [1]Institute of Biotechnology, RWTH Aachen University, Worringer Weg 3, 52074 Aachen, Germany, [2]Institut für Anorganische Chemie, RWTH Aachen University, Landoltweg 1, 52074 Aachen, Germany, [3]DWI - Leibniz Institute for Interactive Materials e.V., Forckenbeckstr. 50, 52056, Aachen, Germany and [4]Institute of Bio- und Geosciences, IBG-1: Biotechnology, Forschungszentrum Jülich GmbH, 52425 Jülich, Germany

Email: Ulrich Schwaneberg* - u.schwaneberg@biotec.rwth-aachen.de

* Corresponding author

[#] Shared first authorship

CD spectra of unmodified FhuA ΔCVFtev directly after refolding and after eight weeks, Thioglo® 1 titration and DLS results

Table of content

1. Circular dichroism (CD) spectroscopy of unmodified FhuA ΔCVF^{tev}
2. Thioglo® 1 titration of FhuA ΔCVF^{tev} and Rh-FhuA ΔCVF^{tev}
3. Dynamic light scattering (DLS) analysis
4. References

1. Circular dichroism (CD) spectroscopy of unmodified FhuA ΔCVF^{tev}

Structural integrity of SDS-denatured transmembrane protein FhuA ΔCVF^{tev} was confirmed after refolding by dialysis against sodium phosphate buffer with the addition of 1 mM EDTA and 50 mM 2-methyl-2,4-pentanediol (MPD) as refolding agent. It was shown that FhuA ΔCVF^{tev} was refolded and folding is kept up to eight weeks.

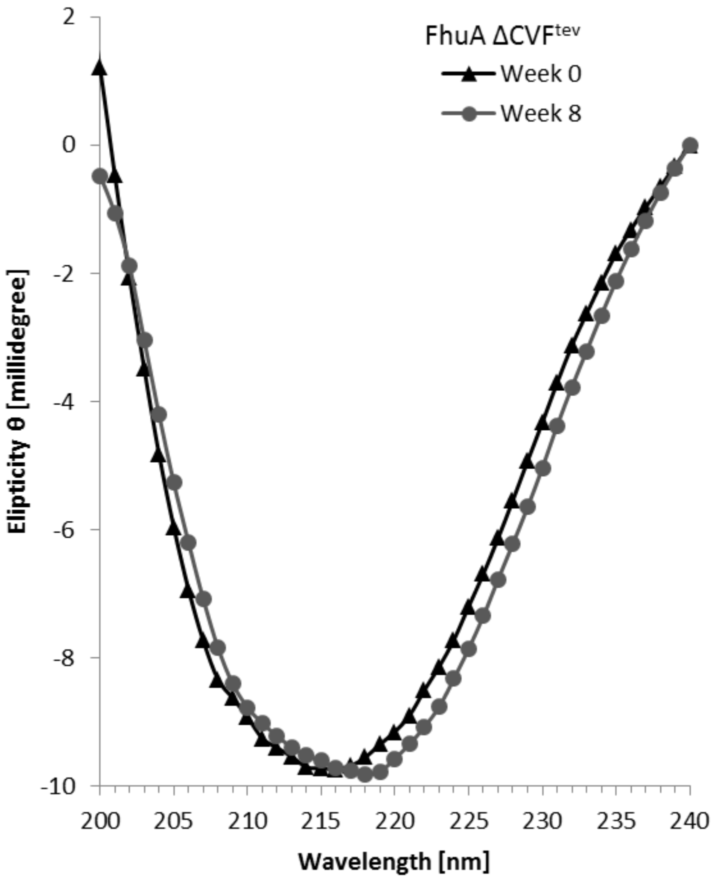

Figure S1: CD spectroscopy of FhuA ΔCVF^{tev} in presence of 2-methyl-2,4-pentanediol as refolding agent after A) refolding and B) after eight weeks. The minimum around 215 nm [1] indicates a proper folding of the transmembrane protein in both cases.

2. Thioglo® 1 titration of FhuA ΔCVF^{tev} and Rh-FhuA ΔCVF^{tev}

There are in total five cysteine residues in the protein sequence of FhuA ΔCVF^{tev}. Four cysteine residues are involved in disulfide bonds according to original amino acid sequence of FhuA. A freely accessible cysteine residues was introduced at amino acid position 545 of FhuA ΔCVF^{tev} (based on PDB id: 1BY3 [2], FhuA wildtype) as previously published [3-4]. The presence of the catalyst in **2** linked to Cys545 was confirmed by decreased Thioglo® 1 fluorescence compared to **1** [3-5].

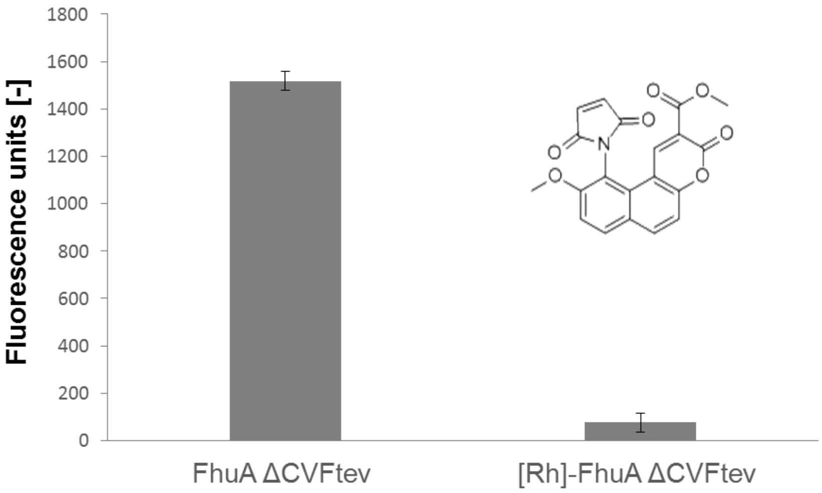

Figure S2: Titration of cysteines of FhuA ΔCVF^{tev} in the presence of 2-methyl-2,4-pentanediol with the fluorescent thiol reagent Thioglo® 1.

3. Dynamic light scattering (DLS) analysis

To show the interaction of the phenylacetylene with the detergent polyethlyene polyethyleneglycol (PE-PEG), DLS measurements were performed (Figure S3).

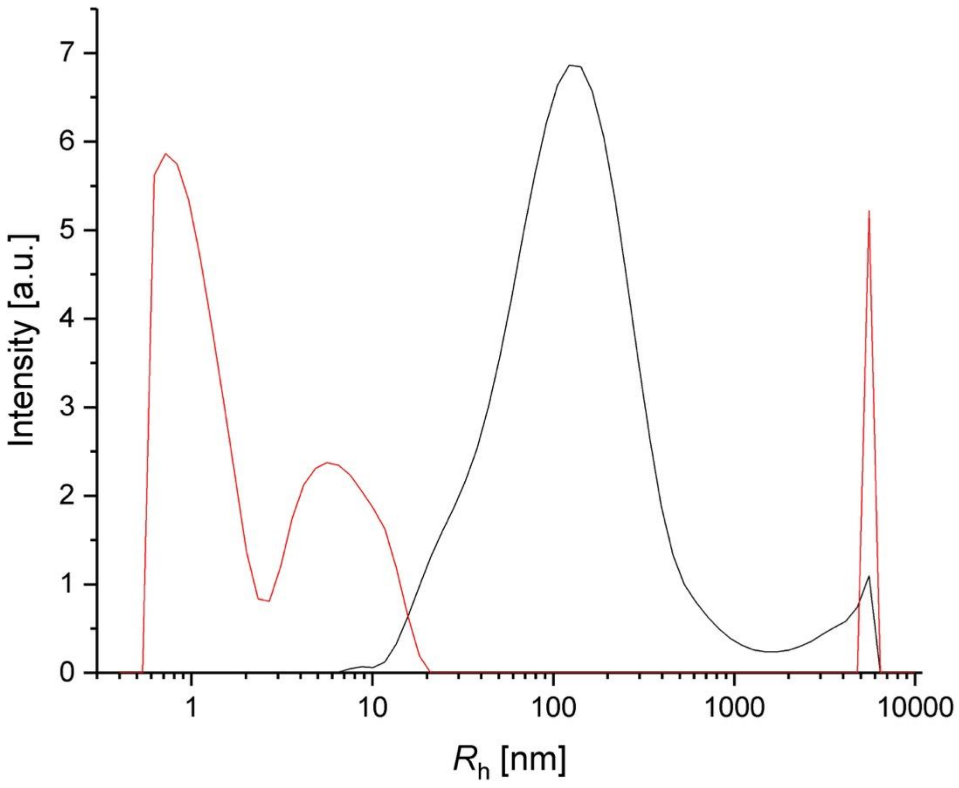

Figure S3: Dynamic light scattering measurements of PE-PEG buffer (black) and PE-PEG buffer containing phenylacetylene (red). PE-PEG, polyethlyene polyethyleneglycol

A clear distortion of the micelles formed by PE-PEG is observed upon the addition of phenylacetylene. This effect is related to the observed protein precipitation when PE-PEG is used as refolding reagent.

4. References

1. Johnson, W. C., Jr., *Annual review of biophysics and biophysical chemistry* **1988**, *17*, 145-166.
2. Locher, K. P., Rees, B., Koebnik, R., Mitschler, A., Moulinier, L., Rosenbusch, J. P., Moras, D., *Cell* **1998**, *95*, 771-778.
3. Philippart, F., Arlt, M., Gotzen, S., Tenne, S. J., Bocola, M., Chen, H. H., Zhu, L. L., Schwaneberg, U., Okuda, J., *Chem-Eur J* **2013**, *19*, 13865-13871.
4. Sauer, D. F., Bocola, M., Broglia, C., Arlt, M., Zhu, L. L., Brocker, M., Schwaneberg, U., Okuda, J., *Chem-Asian J* **2015**, *10*, 177-182.
5. Wright, S. K., Viola, R. E., *Analytical biochemistry* **1998**, *265*, 8-14.
